# The randomised clinical trial and the hazard ratio – medical research’s Emperor’s New Clothes?

**DOI:** 10.1186/1471-2407-14-260

**Published:** 2014-04-14

**Authors:** Richard Stephens, David Stewart

**Affiliations:** 1 Retired, previously MRC Clinical Trials Unit, London, UK; 2 Division of Medical Oncology, The University of Ottawa, Ottawa, Canada

**Keywords:** Randomised clinical trials, Hazard ratio, Individualized treatment, Targeted therapy, Predictive analyses

## Abstract

As the enthusiasm for individualized treatment and targeted therapies continues to gain momentum, it seems timely to re-assess whether our current research tools are fit for purpose. Randomized Clinical Trials compare groups of patients, the Hazard Ratio is a ‘group summary statistic’, and modeling shows that the same Hazard Ratio score could result from a number of scenarios. Thus the current tools do not provide definitive information as to how to treat an individual patient. We therefore need to concentrate on the use of predictive factor analyses to identify the characteristics of subgroups of patients who respond to specific treatments.

## Background

Ever since the first trials of streptomycin for tuberculosis in the 1940’s, the randomized clinical trial (RCT) has been regarded as the gold standard method for assessing new treatments. Similarly, for RCTs with time-to-event outcomes such as survival or progression-free survival, the widely accepted summary statistic to compare treatment arms is the Hazard Ratio (HR), which essentially compares the areas under the survival curves for the 2 treatments. Nevertheless, it is easy to forget that RCTs compare groups of patients, and that the HR is a ‘group summary statistic’ and thus neither RCTs nor HRs provides definitive information as to how to treat an individual patient.

## Discussion

While quality of life, toxicity and cost are often accepted as important secondary outcomes, the common assumption in most cancer RCTs seems to be that the new treatment should be adopted as the new standard for all patients if statistical assessment of relevant time-to-event HR is significantly better than the standard control treatment.

However, this is a false assumption, as the value of a HR can arise from numerous scenarios. For example a HR of 0.75 will be generated if, in an RCT:

• the survival of all patients in the new treatment group is increased by 25%, or

• 25% of patients in the new treatment group experience an approximate 3-fold survival benefit, but the remaining 75% have no survival benefit, or

• 25% of patients in the new treatment group experience an approximate 4-fold survival benefit, but the remaining 75% experience a 10% detriment,

This creates a major dilemma, as it appears impossible to tease out the components of a HR, and distinguish which new treatments should be introduced into routine clinical practice for all patients, and which might actually be detrimental to the majority of patients. None of the possible solutions seem to help: modeling suggests that the survival plots resulting from these various scenarios are virtually indistinguishable, this uncertainty is not ameliorated by increasing the sample size (thus meta-analyses are equally unhelpful), and if predictive factor analyses are undertaken and a subgroup of patients is found that benefits from the new treatment, it is not possible to tell whether that subgroup in turn may need to be subdivided further.

Outcomes such as response can identify the impact of treatment on individual patients, but simply comparing the numbers of patients who respond in an RCT does not overcome the underlying problems, as:

• the RCT alone does not tell us which specific patient subgroups benefit

• different subgroups of patients may benefit from different treatments,

• response rates of combination therapy cannot differentiate between the effectiveness of the individual drugs.

Stewart and Kurzrock [1] have highlighted many of the problems with RCTs in trying to identify ‘who benefits?’ and argued that we need to identify predictive biomarkers for response in phase I and II studies, and use this information to enrich RCTs. Whilst this increases the chances of a clearer outcome, it does not guarantee that all patients will benefit, and does not negate the need to explore other factors over and above the target biomarker. Indeed, if a clear benefit is found in phase I and II studies, there seems little point in running a large expensive RCT.

## Summary

As it is widely acknowledged that the future lies in individualizing treatment, whether it be with new targeted agents or chemotherapy, now may be the time to stand up and expose the RCT and the HR as being as ineffective as the Emperor’s New Clothes in this pursuit, as their past use may have contributed to us discarding many useful treatments, or giving many patients suboptimal treatment. Instead we need to concentrate on the use of predictive factor analyses to identify the characteristics of subgroups of patients who respond to specific treatments. This would require identifying and collating extensive baseline clinical and biological data (from within or outwith RCTs and/or audits) from large numbers of patients who have received the same treatment, perhaps relegating RCTs to a role of supplementary analyses if different treatments appear to give similar response rates in similar subgroups of patients.

## Competing interests

The authors declare that they have no competing interests.

## Authors’ contributions

RS drafted the initial paper, and DS revised and approved the final manuscript. Both authors read and approved the final manuscript.

## Pre-publication history

The pre-publication history for this paper can be accessed here:

http://www.biomedcentral.com/1471-2407/14/260/prepub
